# *Klebsiella pneumoniae* liver abscess in an adolescent with diabetes: A case report

**DOI:** 10.1097/MD.0000000000047847

**Published:** 2026-03-06

**Authors:** Yu-Shin Peng, Lung-Huang Lin

**Affiliations:** a Department of Pediatrics, Cathay General Hospital, Taipei, Taiwan; b Department of Pediatrics, School of Medicine, Fu-Jen Catholic University, New Taipei City, Taiwan.

**Keywords:** diabetes mellitus, *Klebsiella pneumoniae* liver abscess, pediatric, pyogenic liver abscess

## Abstract

**Rationale:**

*Klebsiella pneumoniae* liver abscess is a serious infectious disease that primarily affects patients with diabetes mellitus (DM). The management of Klebsiella pneumoniae liver abscess has been well documented in adults, but pediatric reports remain sparse.

**Patient concerns:**

A 13-year-old girl presented with a history of poorly controlled type 1 DM. She had a high fever for 1 day. Physical examination revealed hepatomegaly with tenderness but without icteric sclera or skin jaundice. Leukocytosis with left shift, elevated CRP, and hyperbilirubinemia were also observed.

**Diagnoses:**

Imaging studies, including abdominal computed tomography and ultrasound, showed a liver abscess. Culture of the pus confirmed *K pneumonia* (KP). Abdominal ultrasound showed a 6 × 5 cm multilocular cystic lesion in liver segment 6 consistent with the diagnosis of pyogenic liver abscess.

**Interventions:**

The patient received intravenous ceftriaxone and metronidazole for 4 weeks, followed by 4 weeks of oral antibiotic therapy with cefixime. Ultrasound-guided percutaneous catheter aspiration was also successfully performed.

**Outcomes:**

After 2 months of antibiotic treatment, the liver abscess shrank significantly, which was also confirmed by follow-up imaging.

**Lessons:**

This case review and literature review highlight the predominance of KP in pediatric pyogenic liver abscesses in Taiwan and the important role of diabetes in pediatric suppurative liver abscess. Children with DM are more likely to develop cryptogenic gas-producing abscesses and metastatic complications. Early diagnosis, timely intervention, and strict glycemic control are essential to improving the prognosis of this high-risk group.

## 1. Introduction

Pyogenic liver abscess (PLA) is a rare but potentially life-threatening pediatric infection. Although it accounts for most liver abscesses in children, its incidence remains low. The incidence of PLA ranges from 3 to 25 per 1,00,000 pediatric hospitalizations worldwide and 8.3 per 1,00,000 pediatric hospitalizations in Taiwan, with no seasonal variation.^[1-3]^

Since the 1980s, *Klebsiella pneumoniae* (KP) has replaced *Escherichia coli* as the major pathogen in East Asia.^[4,5]^ Klebsiella pneumoniae liver abscess (KPLA) is considered a unique invasive syndrome with severe complications, including bacteremia and sepsis metastasis.^[6,7]^

Although the overall mortality rate from KP infection can be as high as 31%,^[8]^ studies on KPLA have shown that the mortality rate can be reduced with timely intervention.^[7,8]^ Diabetes mellitus (DM), an important pediatric risk factor, increases the risk of KPLA and worsening prognosis.^[1,7,9]^ This case report and literature review focused on KPLA in Taiwanese children, particularly those with DM, describing the differences in clinical features, microbiology, imaging, treatment, and prognosis.

## 2. Consent for publication

Ethical approval was obtained from the Institutional Review Board of Cathay General Hospital (IRB No. CGH-P11338). Written informed consent was obtained from the patient for the publication of this case report and accompanying images.

## 3. Case presentation

### 3.1. Patient history

A 13-year-old girl was diagnosed with type 1 DM at 2 years of age. At admission, her height was 142.5 cm, weight was 37.8 kg, and body mass index (BMI) was 18.6 kg/m^2^. From diagnosis until 12 years of age, her diabetes was well-controlled with only 2 episodes of diabetic ketoacidosis (DKA). However, starting at age 12, her disease control deteriorated significantly, with 9 episodes of DKA occurring over the subsequent year prior to the current presentation. These recurrent DKA episodes were frequently associated with infections, including 2 episodes of urinary tract infection with Group B β-hemolytic Streptococcus cultured from urine, and 4 episodes of bronchopneumonia without identified pathogens. The current liver abscess represented her first hepatic infectious complication. This clinical deterioration coincided with the transition to junior high school, where irregular meal timing and academic stress led to inconsistent insulin administration, which was suspected to contribute to the frequent DKA episodes.

### 3.2. Insulin therapy

The patient was receiving conventional insulin therapy with insulin aspart 12 units before each meal (breakfast, lunch, and dinner) and insulin degludec 12 units at bedtime. This regimen had been stable for 1 year prior to admission, with insulin doses gradually increased since her initial diagnosis at 2 years of age.

### 3.3. Glycemic control

Her glycemic control showed progressive deterioration over the 2 years preceding the liver abscess, with hemoglobin A1c (HbA1c) levels ranging from 10.8% to 13.7% between ages 11 and 12 years. On admission, her fasting blood glucose was 445 mg/dL. By the third day of hospitalization, fasting blood glucose improved and stabilized between 110 and 250 mg/dL. During the 2-year follow-up period after resolution of the liver abscess, her HbA1c ranged between 9% and 11%. Continuous glucose monitoring was not utilized in this patient’s care.

### 3.4. Laboratory findings

Lipid profile over the 5 years prior to admission showed total cholesterol levels ranging from 175 to 185 mg/dL, high-density lipoprotein cholesterol from 65 to 95 mg/dL, low-density lipoprotein cholesterol from 75 to 130 mg/dL, and triglycerides progressively increasing from 55 to 200 mg/dL.

Liver function tests over the same 5-year period demonstrated alanine aminotransferase levels between 10 and 50 IU/L and aspartate aminotransferase levels between 18 and 50 IU/L. Coagulation studies showed prothrombin time ranging from 12 to 14 seconds and international normalized ratio between 1.1 and 1.2. At the time of admission for liver abscess, alanine aminotransferase was 32 IU/L, aspartate aminotransferase was 45 IU/L, and direct bilirubin was 0.1 mg/dL. Total bilirubin and albumin were not measured.

Despite her prolonged history of poorly controlled diabetes, the patient had no evidence of diabetic microvascular complications, including diabetic retinopathy, nephropathy, or neuropathy.

### 3.5. Clinical presentation

**Figure 1.**
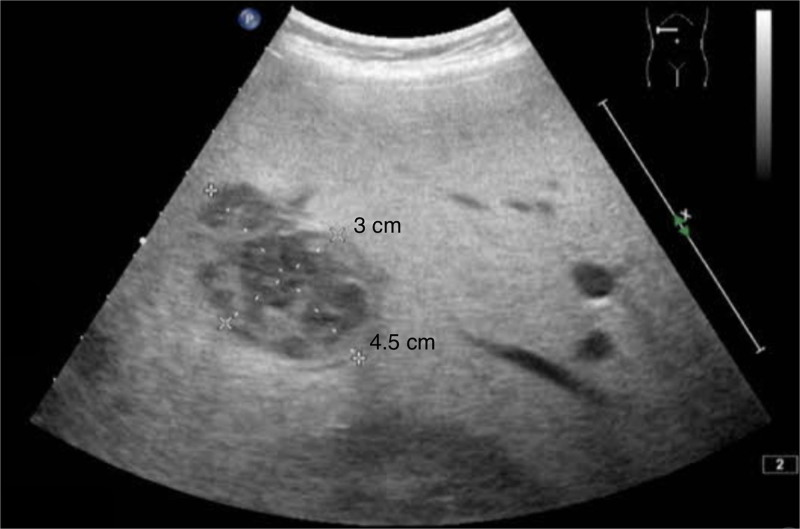
Abdominal ultrasound showing 3 × 4.5 cm multilocular cystic lesion in liver segment 6.

**Figure 2.**
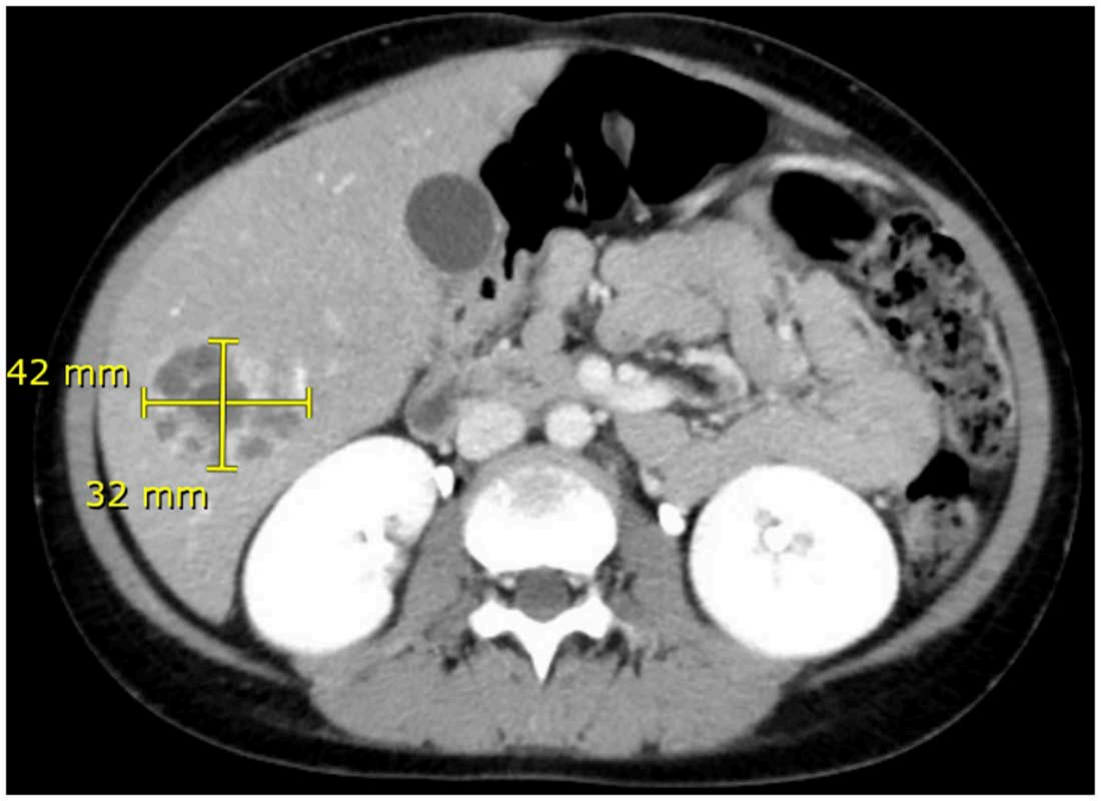
Cross-sectional abdominal CT showing 3.2 × 4.2 cm multilocular cystic lesion in liver segment 6. CT = computed tomography.

**Figure 3.**
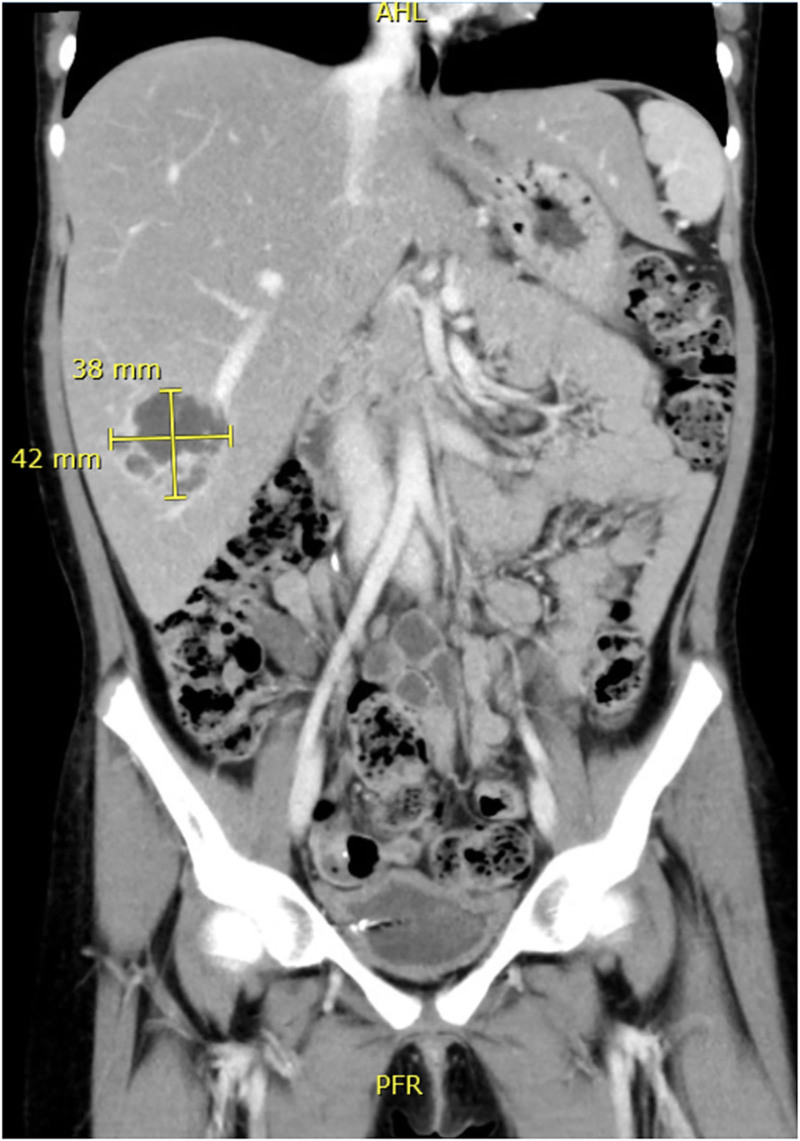
Coronal abdominal CT scan of the abdomen showing 3.8 × 4.2 cm multilocular cystic lesion in liver segment 6. CT = computed tomography.

The patient presented with fever (38.9°C) for 1 day without other symptoms. Physical examination revealed tender hepatomegaly without scleral or cutaneous jaundice. Blood cultures were negative. Abdominal ultrasound (Fig. 1) and computed tomography (CT) scan (Figs. 2 and 3) showed a multilocular cystic lesion of approximately 3 × 4 cm in liver segment 6, consistent with a diagnosis of liver abscess.

### 3.6. Treatment and follow-up

**Figure 4.**
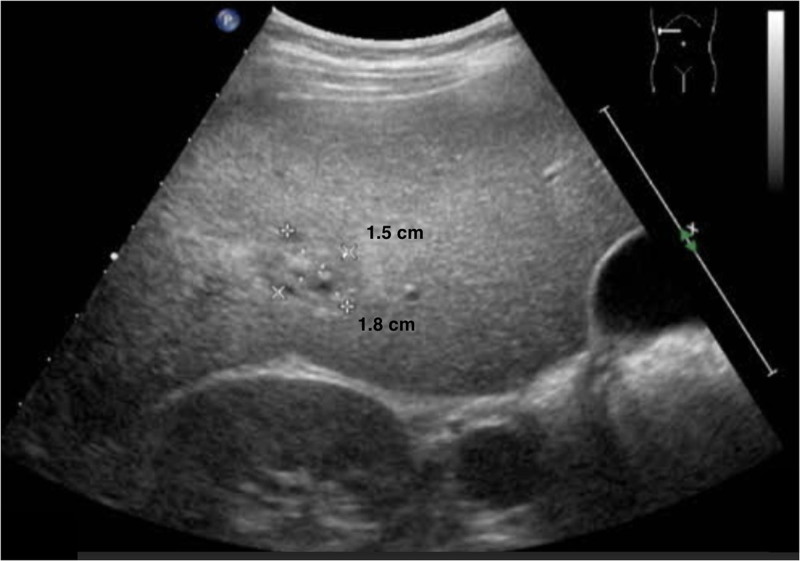
Abdominal ultrasound showing 1.8 × 1.5 cm residual lesion in liver segment 6.

The patient underwent a single ultrasound-guided aspiration of 4 mL turbid, yellowish pus. The aspirate cultured KP, which was sensitive to all antibiotics tested. The patient received intravenous ceftriaxone and metronidazole for 4 weeks, followed by oral cefixime for 4 weeks. Follow-up ultrasound after the end of antibiotic therapy showed interval resolution of the abscess, with a residual lesion in segment 6 measuring 1.8 cm × 1.5 cm (Fig. 4).

## 4. Discussion

This literature review is based on our pediatric case from Taiwan. Here, we describe the clinical presentation of PLAs, with a particular focus on cases with DM.

### 4.1. Pathogen

PLA should be included in the differential diagnosis of liver masses, especially in patients with sepsis.^[9]^

The causes of bacterial liver abscess are diverse and often involve infections originating from the gastrointestinal tract or intra-abdominal cavity, or from postoperative complications.^[1]^ The pathogens usually invade the liver via blood circulation or ascending infection of the biliary tract.^[10]^ Unlike Western countries where the most common pathogen is *E coli*, KP is the most common pathogen of liver abscesses in Taiwan, especially in patients with DM (i.e., in 70–78%).^[1,4,5,9,11]^ Over the past decades, this clinical syndrome has become well-recognized in Taiwan, with liver abscesses caused predominantly by a single microorganism, KP.^[11]^

### 4.2. Relation with diabetes mellitus

KPLA was first identified in Taiwan in 1986, and current evidence supports its designation as a distinct, aggressive syndrome that is often associated with metastatic sepsis and increased disease severity.^[5,7,11,12]^ In pediatric patients, DM is a significant risk factor for the development and progression of liver abscesses. The incidence of cryptogenic and gas-producing liver abscesses is higher in pediatric patients with DM, both of which are associated with a severe clinical course and poor prognosis.^[3,7,8,13,14]^ These findings are consistent with existing evidence that hyperglycemia impairs neutrophil function, particularly against encapsulated pathogens (e.g., KP serotypes K1 and K2), which are known to resist phagocytosis and evade host immunity.^[5,6]^

The association between KPLA and diabetes is particularly strong, with DM being a frequent underlying condition that predisposes patients to the development of liver abscess with this organism.^[15]^ When patients with KPLA are compared with those who have polymicrobial liver abscess, significantly higher incidences of diabetes or glucose intolerance (75% vs 4.5%) have been observed.^[11]^ Moreover, liver abscess with KP is closely linked to poor glycemic control.^[14,16]^ Although PLA is traditionally considered a rare complication of diabetes, usually seen in adults >50 years of age who have had diabetes for many years, recent reports suggest that occult PLA should be considered in children and adolescents with fever of unknown origin and diabetes, particularly in those with prolonged periods of poor glycemic control.^[14,16]^ In our case, the patient’s extremely poor glycemic control (HbA1c 10.8–13.7%) and recurrent episodes of DKA likely contributed to the development of this serious infectious complication.

Within the past 30 years, hypervirulent variants of Klebsiella *pneumoniae* (hvKp), which emerged in the Asian Pacific Rim, have spread globally.^[16]^ These hvKp strains are capable of causing life-threatening PLAs, endophthalmitis, pneumonia, central nervous system infection, and bacteremia, even in healthy individuals.^[16]^ Although hvKp-caused PLAs have been seldom reported in pediatric populations, awareness of this potentially fatal complication in adolescents with DM is crucial.^[16]^

### 4.3. Presentations

That most children presented with fever, abdominal pain, and vomiting is consistent with previous reports from Taiwan and other endemic areas.^[2,3,13]^ However, less than half of these patients meet the Fontan triad, suggesting nonspecific clinical manifestations of liver abscess in children. Some patients also have chest symptoms such as cough and pleural effusion, suggesting the possibility of subphrenic irritation or early pulmonary dissemination, which may affect initial diagnosis.^[3]^

### 4.4. Labs and imaging

Hematologic examinations reveal leukocytosis, elevated C-reactive protein, and abnormal glutamic oxaloacetic transaminase or glutamic pyruvic transaminase, bilirubin, and alkaline phosphatase.^[17]^ Liver abscess diagnosis relies primarily on imaging studies. Abdominal ultrasound has a diagnostic sensitivity of 80% to 90%, while enhanced CT scanning has higher sensitivity and specificity, with reported diagnostic accuracy exceeding 95%.^[2]^ In patients with DM, CT scanning is particularly valuable for early detection of high-risk features like gas formation, which is associated with rapid clinical deterioration and septic shock.^[3]^

### 4.5. Treatment

Blood cultures have a lower positive rate compared with pus cultures, which are more informative for identifying KP and Streptococcus, the 2 most commonly isolated pathogens according to our literature review.^[1,13,18,19]^ This highlights the importance of timely drainage procedures to guide targeted antimicrobial therapy.^[7]^ In Taiwan, KP has been emerging as the leading cause of liver abscess, and most patients with KPLA are diabetics without preexisting biliary diseases.^[19]^

PLA is primarily treated with a combination of intravenous antibiotics and percutaneous catheter drainage (PCD), reflecting the current clinical trend toward minimally invasive approaches over surgical intervention.^[2,3,7,20]^ The average duration of parenteral antibiotic therapy is approximately 3 weeks, followed by 3 to 6 weeks of oral antibiotic therapy.^[1]^ Surgical intervention is reserved for patients with ruptured abscesses, inadequate response to PCD, or concomitant abdominal lesions.^[3,13,21]^ These results are consistent with previous Taiwanese pediatric series, including early studies from southern Taiwan and studies as early as the 1990s, which demonstrated a shift from surgery to minimally invasive techniques and an overall improvement in treatment outcomes.^[2,13]^

### 4.6. Prognosis

Of note, PLA in immunocompromised hosts (e.g., patients with cirrhosis) has also been associated with adverse outcomes in adult studies, suggesting that host defense plays a central role in all age groups.^[17]^ In our pediatric case, DM status served as a surrogate marker for immunosuppression, highlighting the importance of glycemic control as part of a comprehensive treatment strategy. Extrahepatic metastases, such as septic endophthalmitis, often occur with serious complications, particularly in patients with diabetes.^[15]^ The association between KPLA and diabetes is so close that a search for underlying DM is warranted in all patients with KPLA.^[15]^

Some limitations must also be acknowledged. This retrospective case report has potential biases in data completeness and symptom recording. In addition, microbiological and serotype confirmation of KP strains (e.g., K1/K2, rmpA) was not performed uniformly, limiting pathogen-specific analysis. Despite these limitations, this report is one of the most comprehensive pediatric reviews of KPLA in Taiwan and provides clinically meaningful insights into the risk stratification and treatment of liver abscesses in children with DM.

## 5. Conclusion

This report highlights the clinical and microbiological status of PLA in Taiwanese children, in which KP has emerged as the main pathogen and for which DM is a major risk factor. Regardless of abscess size, children with DM are more likely to develop cryptogenic abscesses, gas-producing abscesses, and metastatic complications. Early recognition, timely imaging, evidence-based antibiotic therapy, and PCD remain the treatment cornerstones. For high-risk groups such as children with DM, strict glycemic control should be considered an important component of treatment to enhance the immune response and reduce the risk of spread.

## Author contributions

**Writing – original draft:** Yu-Shin Peng.

**Writing – review & editing:** Lung-Huang Lin.
